# Development and Validation of the Psychological Abuse in Relationships Scale

**DOI:** 10.1177/08862605251325912

**Published:** 2025-04-03

**Authors:** Elizabeth Veronica-Mary McLindon, Cynthia Brown, Mandy McKenzie, Laura Tarzia, Kelsey Hegarty

**Affiliations:** 1 The University of Melbourne, VIC, Australia; 2 The Royal Women’s Hospital, Melbourne, VIC, Australia

**Keywords:** intimate partner violence, psychological violence, coercive control, scale, measurement

## Abstract

Psychological abuse within intimate relationships is a highly prevalent subtype of intimate partner violence (IPV) that is frequently associated with other types of IPV such as sexual or physical violence. Psychological abuse can cause enduring harm, including the loss of agency and self-belief, and entrapment in a relationship. Previous measures of psychological abuse have been characterized by inconsistencies in conceptualization and measurement contributing to problems in identifying its prevalence, impact, and patterns. To address many of the existing challenges and gaps, we developed a new measure of psychological abuse, building upon scale development work to date, and in consultation with lived experience and academic experts. A sample of 765 adult women in Australia completed our survey of 31 psychologically abusive behaviors to establish reliability and validity evidence for a new measure of psychological abuse victimization and impact. *Exploratory factor analysis* generated a scale comprising 20 items and four factors—Severe Psychological Abuse, Coercive Emotional Abuse, Restrictive Isolating Abuse, and Financial Abuse—with Cronbach’s alphas ranging from 0.73 to 0.85 and overall explained variance of 57.2%. Exhibiting evidence of validity and reliability, the Psychological Abuse in Relationships Scale is a contemporary, concise, and comprehensive measure of psychological IPV that will improve the ability of researchers to identify this common and harmful type of abuse.

## Introduction

Intimate partner violence (IPV), a major health and social issue, is defined as harmful physical, sexual, or psychological behavior by a partner or ex-partner (World Health Organization [WHO], 2013). IPV is associated with acute and chronic bio-psychosocial health problems (Bacchus et al., 2018; Mellar et al., 2023), and premature death (Australian Domestic and Family Violence Death Review Network & Australia’s National Research Organization for Women’s Safety (ANROWS), 2022). While IPV can occur in any type of intimate relationship, it is most commonly perpetrated by men against women and children and is underpinned by inequitable gender roles and misogynistic attitudes (Flood & Pease, 2009; Heise, 2011).

IPV is often separated into three subtypes: physical, sexual, and psychological. Psychological abuse may be subtle and difficult to identify, characterized by ongoing patterns of emotionally and verbally abusive behaviors, such as degrading, belittling, and humiliating statements, and controlling or coercive behaviors, including threats, isolation, and monitoring (Beckwith et al., 2023; WHO, 2021). Research with men who have used psychological abuse suggests that their aim is to control a victim-survivor (hereafter referred to as “survivor”), through diminishing their sense of self-worth and creating dependence, fear, and mental anguish (Follingstad, 2007; Stark, 2007; Tolman, 1989). Dokkedahl et al. (2019) theorize psychological abuse as a set of behaviors occurring along a continuum of increasing severity, from verbally aggressive behaviors, to control and isolation. Psychological abuse may precede or co-occur with other types of violence, acting as an essential agent in an environment where physical and sexual IPV is introduced and maintained (Hegarty et al., 2005; Stark, 2007; Tarzia & Hegarty, 2023).

Psychological abuse appears to be the most prevalent subtype of IPV (WHO, 2021), and global estimates suggest that two in five (43%) women have experienced psychological abuse (European Union Agency for Fundamental Rights, 2014; Stöckl et al., 2021). Gender is one of many intersecting inequalities (along with disability, socioeconomic factors, age, race, among others) that increases the risk of psychological abuse and influences the lived experience of survivors (Beckwith et al., 2023). While psychological abuse may have initially been of interest to researchers and clinicians as a risk factor for later physical IPV, survivors have consistently identified psychological abuse as a particularly insidious and harmful type of IPV in its own right (Dokkedahl et al., 2022), with effects perhaps more injurious and lingering than physical abuse (Follingstad, 2007). Survivors speak of a loss of agency, autonomy, self-trust, and self-belief, leading to a feeling of entrapment (ANROWS, 2021; Beckwith et al., 2023). A recent meta-analysis found the main mental health impacts of psychological abuse are posttraumatic stress disorder, depression, and anxiety, with coercive and controlling behaviors most strongly associated with posttraumatic stress and emotional, verbal, dominating, and isolating behaviors most strongly associated with depression (Dokkedahl et al., 2022).

Coercive control is defined as a pattern of manipulating, dominating, and controlling tactics used with the intent of preventing agency and autonomy and including a range of psychologically, sexually, and physically abusive behaviors (Stark, 2007; Stark & Hester, 2019; Winstok & Sowan-Basheer, 2015). Stark and Hester (2019) contend that coercive control is not a type of abuse but a motive for engaging in abusive behaviors and a climate created within abusive relationships. Nevertheless, the term coercive control is sometimes used as a substitute for psychological abuse (Boxall & Morgan, 2021), positioned as a type of IPV, or as a subcategory of psychological abuse (WHO, 2021). Stemming from the continuum of violence that typifies psychological abuse, differences in how it is conceptualized in research and clinical practice abound (Dokkedahl et al., 2019; WHO, 2021). This has contributed to psychological abuse remaining an indistinct concept despite many attempts at the definition (Beckwith et al., 2023; Dokkedahl et al., 2022; Winstok & Sowan-Basheer, 2015). For behavior to be deemed abusive, most definitions are conditional upon the behavior having the potential to cause harm and being used with the intention to cause harm (Winstok & Sowan-Basheer, 2015).

The diverse conceptualizations of psychological abuse have led to methodological and measurement issues (Dokkedahl et al., 2019). Available psychological abuse measures contain multiple inconsistencies, including between *types* of behaviors included (Dokkedahl et al., 2022; WHO, 2021). For example, some surveys measure controlling behaviors as part of a broader pattern of psychological abuse, and some measure controlling behaviors separately (WHO, 2021). Economic abuse is frequently measured independently from other types of non-physical abuse, and less commonly, so is stalking (WHO, 2021). Measurement disparities include if/how behavior *frequency* is measured and whether/how behavior *severity* is addressed (Dokkedahl et al., 2022; WHO, 2021). Other problems contributing to poor psychological abuse measurement include if/how to set *thresholds* for determining prevalence (Follingstad, 2007; Heise et al., 2019), the total *number of items* in a measure, whether psychological abuse is the sole or partial *focus* of a scale, and terminology variations (Winstok & Sowan-Basheer, 2015). In a review of 21 psychological violence measures, Dokkedahl et al. (2019) found these variations made it difficult to compare the effects of psychological abuse on mental health across studies. The WHO (2021) cites the lack of agreed psychological abuse measures to be one of the key remaining challenges in prevalence research.

We uncovered similar methodological issues in a review of 31 existing measures of psychological abuse (Australian Bureau of Statistics, 2016; Borrajo et al., 2015; Boxall & Morgan, 2021; Brown & Hegarty, 2021; Campbell et al., 2009; Dutton et al., 2005; Family Safety Victoria, 2018; Follingstad, 2011; Follingstad et al., 2005; Ford-Gilboe et al., 2016; Foshee et al., 1998; García-Moreno et al., 2005; Graham-Kevan & Archer, 2003; Hegarty et al., 2005; Hudson & Mclntosh, 1981; Johnson et al., 2014; Maldonado et al., 2020; McCauley et al., 2017; Murphy & Hoover, 1999; Rodenburg & Fantuzzo, 1993; Rogers & Follingstad, 2014; Sackett & Saunders, 1999; Shepard & Campbell, 1992; Sherin et al., 1998; Smith et al., 2002; Straus et al., 1996; Sullivan & Bybee, 1999; Swahnberg & Wijma, 2003; Tolman, 1989, 1999; Upadhyay et al., 2014). Issues included: gaps in the dimensions covered (e.g., Sackett & Saunders (1999) do not include items about isolation, intimidation or humiliation); the use (e.g., Murphy & Hoover, 1999), or not (e.g., Foshee et al., 1998), of subscales; if/how behavior impact (e.g., Rodenburg & Fantuzzo, 1993), or severity (e.g., Follingstad, 2011), is collected; if/how incidence is assessed (e.g., via a Likert-type scale as per Shepard & Campbell, 1992 etc); varying recall periods (e.g., in the past 3 months (McCauley et al., 2017), 6 months (Tolman, 1999), 12 months (Maldonado et al., 2020), or adult lifetime (García-Moreno et al., 2005); and the use of thresholds (e.g., Hegarty et al., 2005) versus continuums (e.g., Ford-Gilboe et al., 2016). Some measures were brief (e.g., Swahnberg & Wijma, 2003 is 13 items) or prohibitively long (e.g., Tolman, 1999 is 58 items); contained now outdated language (e.g., Hudson and Mclntosh, 1981) or had a focus on the actions of people using violence (e.g., Straus et al., 1996), among many other issues.

The present study builds upon conceptual and measurement work in the field by developing and validating a new, concise, and comprehensive measure of psychological abuse. The aim of this study was to develop and validate a new measure of psychological abuse victimization behaviors—and their emotional impacts—among a diverse and nationally representative sample of women in Australia.

## Methods

Following evidence-based recommendations (Pett et al., 2003), scale development occurred over three phases and built upon existing work in the psychological abuse measurement field (Follingstad, 2011; Tolman, 1989). Phase 1 involved the generation of scale items; Phase 2, the continuation of item adaptation, testing, and piloting the measure in addition to generating evidence of face and content validity; and Phase 3 encompassed initial construct validation of the new scale.

### Phase 1—Item Generation and Construct Formation

To compile a comprehensive list of psychologically abusive behaviors experienced by women, items from the above-mentioned 31 measures about psychological abuse were reviewed for inclusion. Items pertaining to specific methods of perpetrating psychological abuse (i.e., via a technological device) were not included (see Brown & Hegarty, 2021 for recently validated measure of technology-facilitated abuse). More than 530 items spanning a wide range of behaviors were thematically grouped into distinct types of psychological abuse. All authors discussed and agreed upon items deemed the best example of each type of psychological abuse, resulting in a list of behaviors spanning the severity spectrum (Australian Bureau of Statistics, 2016; Brown & Hegarty, 2021; Family Safety Victoria, 2018; Follingstad, 2011; Follingstad et al., 2005; García-Moreno et al., 2005; Graham-Kevan & Archer, 2003; Hegarty et al., 2005; Johnson et al., 2014; Rodenburg & Fantuzzo, 1993; Sackett & Saunders, 1999; Sullivan & Bybee, 1999; Tolman, 1999). The language used across the items in the measure was updated so that it was behavioral, first-person past tense, tonally consistent, contemporary, and gender-neutral. In total, 30 items were developed for inclusion in a new measure of psychological abuse.

### Phase 2—Item Generation and Face and Content Validity

Content experts were consulted to conceptually test the list of 30 items and establish face and content validity. Ten national and international academic, policy, and health experts (recruited from the authors’ networks) and five experts with lived experience of psychological abuse (recruited via a University of Melbourne panel) participated in semi-structured discussion groups to identify gaps and redundancies and suggest language changes. This work led to item refinements and improvements, mostly regarding clarity and scope. Items were then piloted with 10 women who had experienced psychological abuse, which resulted in one new item (“threatened to use the legal system against me”) and small changes to the wording of eight items. The final questionnaire consisted of 31 items representing three distinct psychological abuse tactic types: emotional/verbal (6 items), coercion/dominance (12 items), and control/isolation (13).

### Phase 3—Initial Validation of Scale

#### Participants and Procedure

A nationally representative sample was sought, comprised of English-speaking women living in Australia who had been in an abusive adult intimate relationship during the last 5 years. Panel members of an experienced commercial research company (iLink) were sent recruitment information about the nature of the survey and invited to voluntarily opt-in. Participants who completed a survey received an honorarium of panel points and entered a draw to win one of two iPads.

Psychological abuse items were presented to participants within a broader survey conducted online between February 14 and April 5, 2022. The survey asked participants about different types of IPV, their health, service use, and demographic background (Australian Bureau of Statistics, 2017; Breslau et al., 1999; Brown & Hegarty, 2021; Fraser et al., 2014; Hegarty et al., 2005; Hegarty et al., 2022; Kroenke et al., 2009; Rosenberg, 1965; Ware et al., 1998). Participants were eligible for the study if they were female, aged 18 years plus, and answered “*yes*” to at least one of seven screening behaviors having occurred in the last 5 years ( *feeling afraid; having day-to-day activities controlled; isolation from family, friends, or other people; monitoring, manipulative or harassing behaviors; threats to hurt them or others they cared about; being hit, slapped, kicked, or otherwise physically hurt; or attempted pressure/ pressure into unwanted sexual activity*). The survey took approximately 30 minutes to complete and included prompts to guided self-care activities and support information if required.

A group of 1,026 diverse participants completed the full survey. Participants were asked about their experience of 31 psychological abuse items by any partner or ex-partner (of a relationship lasting longer than 1 month) in the last 5 years (*yes/no*). On a Likert-type scale, those who answered “yes” were asked how often each behavior had occurred in the last 12 months (*Not in the past 12 months* [0], *Once* [1], *A few times* [2], *Monthly* [3], *Weekly* [4], *Daily/almost daily* [5]). A sample of 765 participants who had experienced one or more of the 31 psychologically abusive behaviors during the last 12 months were included in the analysis. Since frequency scores are not necessarily an indication of the experience or severity of the behavior, for each 12-month behavior selected, participants were asked how *scared* and *controlled* the action made them feel (*Not at all* [0], *Slightly* [1], *Moderately* [2], *Very* [3], *Extremely* [4]; Brown & Hegarty, 2021).

#### Analysis

Data were analyzed using IBM SPSS 29.0.0.0 for Windows (SPSS Inc., 2007). Factor analysis was used to reduce the list of behavioral items to a practical scale length, understand the structure, and verify the reliability and validity of the new scale (Groth-Marnat, 2009; Pett et al., 2003; Tabachnick & Fidell, 2013). Assessments of missing values and limitations (outliers, normality of distribution, and factorability of the data) were undertaken (Pett et al., 2003; Tabachnick & Fidell, 2013). Common factor analysis was used to develop a measurement instrument that identified the underlying types of the 31 items (Sarstedt & Mooi, 2019; Tabachnick & Fidell, 2013). Alpha factoring extraction with direct obliman oblique rotation was employed (Pett et al., 2003; Tabachnick & Fidell, 2013). For the purpose of scale development, participants were scored as having experienced psychological abuse if they had experienced any of the 31 behaviors at least once in the last 12 months.

## Results

The majority of participants were born in Australia (78.2%), did not identify as Aboriginal or Torres Strait Islander (93.1%), and spoke English at home (89.2%) (Table 1). Three-quarters had a male partner (75.1%), children (67.9%), and were living in a metropolitan area at the time of the survey (64.7%). More than two-thirds of participants had post-secondary school qualifications, and 59.6% were employed in paid work outside the home.

**Table 1.** Participant Demographics and Characteristics (*N* = 765).

Demographic Categories	Frequency	Percent
Age		
18–24 years	90	11.8
25–45 years	336	43.9
>45 years	339	44.3
Gender of partner^a^		
Male	428	75.1
Female	141	24.7
Nonbinary	1	0.2
State of residence		
New South Wales	228	29.8
Victoria	225	29.4
Queensland	162	21.2
Western Australia	66	8.6
South Australia	58	7.6
Tasmania	14	1.8
Australian Capital Territory	11	1.4
Northern Territory	1	0.1
Residential regionality		
Metropolitan	495	64.7
Regional	245	32.0
Not specified	25	3.3
Indigenous status		
Aboriginal	46	6.0
Torres Strait Islander	1	0.1
Aboriginal and Torres Strait Islander	6	0.8
Neither Aboriginal or Torres Strait Islander	712	93.1
Country of birth		
Australia	598	78.2
Other	167	21.8
First language		
English	682	89.2
Other	83	10.8
Highest education level		
Degree or higher degree	266	34.8
Diploma or certificate	277	36.2
Year 12 or lower	222	29.0
Employment status		
Fulltime (35+ hr per week)	226	29.5
Part-time (<35 hr per week)	230	30.1
Not in paid work	309	40.4

a *n* = 570 participants reported being in a current relationship and responded to the partner gender question.

### Assessing Limitations

Data from the 765 participants were screened for errors and cleaned. Due to the Likert scale design, there were no univariate outliers, however, 51 cases were highlighted through anomaly testing. An examination of these cases revealed several with similar responses across multiple items. This was not considered a valid reason for exclusion given the nature of the items and the plausibility of respondents reporting similar behavior patterns. Satisfying the minimum requirement for factor analysis, the sample size of 765 offered a ratio of 24.7 cases per variable (Pett et al., 2003; Tabachnick & Fidell, 2013). An absence of univariate normality was found, with positive skewness, positive non-normal kurtosis, and significant Kolmogorov–Smirnov tests on many variables (Pallant, 2011). Histograms and Q–Q plots also suggested an absence of univariate normality. Univariate non-normality is not uncommon in IPV research (Ryan, 2013), nor considered critical to factor analyses when working with large samples, so a decision was made to review nontransformed factor analyses and re-assess if the solution was uninterpretable (Field, 2009; Oppong & Yao, 2016; Tabachnick & Fidell, 2013).

Inspections of scatterplots suggested departures from linearity. Meeting the assumption of linearity is also not considered critical when the goal is to reduce the number of items and examine correlational patterns between items (Tabachnick & Fidell, 2013), so a further decision was made to review the nontransformed factor solution for interpretability. Following inspection of eigenvalues, the determinant, the correlation matrix, and squared multiple correlations, it was concluded that neither multicollinearity nor singularity was present in the dataset (Field, 2009; Tabachnick & Fidell, 2013).

Examination of the factorability of the 31 items was undertaken. Inspection of the correlation matrix revealed that more than 65% of the correlations between pairs of variables exceeded 0.3. Examination of the significance test of correlations affirmed that all correlations were significant at *p* = .01. Inspection of the anti-image correlation matrix confirmed that more than 99% of the correlations were less than 0.1. The KMO’s measure of sampling adequacy on all variables ranged from 0.941 to 0.978. Collectively, these indicators suggested the dataset was suitable for factor analysis (Field, 2009; Kaiser, 1974; Pett et al., 2003; Tabachnick & Fidell, 2013).

#### Construct Validity

Common factor analysis was undertaken on the 31 items. Multiple extraction methods, including maximum likelihood methods, unweighted least squares, GLS, alpha factoring, and image factoring, were undertaken and preliminarily reviewed (Pett et al., 2003; Tabachnick & Fidell, 2013). The unrotated alpha factoring solution was selected due to it being the most interpretable. An assessment of the cumulative percentage of variance, the scree plot, and eigenvalues greater than 1.00 was undertaken to conclude that four factors should be retained (Pett et al., 2003; Tabachnick & Fidell, 2013). The factors were expected to correlate so oblique rotation solutions (direct obliman, promax, and orthoblique) were examined for the four-factor solution (Pett et al., 2003; Tabachnick & Fidell, 2013). Interpretability, meaning, and scientific utility of the solution were taken into consideration (Pett et al., 2003; Tabachnick & Fidell, 2013), leading to the decision to retain the four-factor solution with direct obliman rotation. After determining the influence of their removal on the scale’s reliability to be inconsequential because of like-items that were retained, two items loading <.3 across all factors (*Got me to pay their bills or debt, Told me I couldn’t take care of myself without them*) and one item loading >.3 on multiple factors (*Threatened to hurt me/others I care about*) were removed.

To interpret the solution, a further four-factor alpha factoring direct obliman solution was run on the remaining 28 items. Scale brevity was deemed important, and a decision was made to further delete items loading <0.45. This led to the removal of an additional 8 items (Table 2), resulting in a final scale of 20 items. Two items loading just below this threshold were retained due to their conceptual importance and impact: *Drove erratically/dangerously when I or my child/ren were in the car* (loading: 0.408) was rated by more participants than any other as making them feel scared, and the item, *Told me I imagined things or denied their own behavior* (loading: 0.442) was rated as causing the majority of participants to feel controlled. A higher proportion of participants rated the items in factor 1 as inducing fear and a feeling of being controlled compared to the other factors, leading to the title: “Severe Psychological Abuse.”

**Table 2.** Items Removed From Scale.

Removed Items
Dominated my personal space Lied about me to other people Punished me by withholding affection or refusing to speak to me Threatened to leave if I didn’t do what they wanted Threatened suicide to control me Threatened to use the legal system against me Expected me to follow certain rules Intimidated or controlled me through looks and signals

*Note*. Items loading <0.45 removed from scale.

From a utility perspective, a scale containing 20 items was considered a suitable and practical length, so the solution was run a final time, revealing four clear factors. In this final solution, the first and second factors exchanged positions in the factor list, but the items continued to load consistently with their original factors. The final items and factor groupings, including their Cronbach’s alphas and explained variances, are shown in Table 3.

**Table 3.** Summary of Final Four-Factor Solution With Reliabilities and Explained Variance.

Factor, Factor Name, Cronbach’s Alpha, Explained Variance, and Items	Loading
Factor 1: Severe psychological abuse, α = .852, Variance explained = 36.646% (7 items)	
Punished or deprived the children to hurt me	0.738
Told me that they would kill me/my children	0.687
Threatened to take the children from me	0.615
Restricted access to essentials of daily living (e.g., food, phones, transport, housing, aids, visa/passport, or spiritual practice)	0.612
Threatened to harm family pets	0.611
Interfered with my health care (e.g., treatment for physical or mental health or alcohol and other drug issues)	0.560
Tried to damage my relationship with my child/ren	0.464
Factor 2: Coercive emotional abuse, α = .734, Variance explained = 8.533% (5 items)	
Frightened me by screaming or breaking things	0.693
Threatened to destroy things	0.565
Put me down or humiliated me in front of others	0.505
Told me I imagined things or denied their own behavior	0.442
Drove erratically/dangerously when I or my child/ren were in the car	0.439
Factor 3: Restrictive isolating abuse, α = .835, Variance explained = 6.497% (5 items)	
Controlled my day-to-day activities (e.g., where I go and what I do)	0.720
Kept track of where I was/who I was with (e.g., GPS tracking, monitoring social media, or phoning me)	0.713
Monitored my day-to-day activities or what I did with my time	0.700
Was jealous and possessive	0.543
Tried to keep me from socializing with family or friends	0.494
Factor 4: Financial abuse, α = .760, Variance explained = 5.577% (3 items)	
Used our money/made important financial decisions without talking to me	0.700
Controlled my access to money	0.634
Took our/my money and made me worry about not having enough	0.582

*Note*. Alpha Factoring extraction method, Direct Obliman rotation method.

To name the factors, items in each factor were reviewed to identify common themes which were then considered in light of the relevant literature. Factor 1 was named Severe Psychological Abuse, Factor 2: Coercive Emotional Abuse, Factor 3: Restrictive Isolating Abuse, and Factor 4: Financial Abuse. Total variance explained by these items was 57.25%, considered acceptable in behavioral science research (Pett et al., 2003). The new scale was titled the Psychological Abuse in Relationships (PAR) Scale.

#### Internal Consistency

The scale produced an overall internal consistency of α = .905. Cronbach’s Alpha’s for the individual factors ranged from .73 to .85. The internal consistency overall and of each item set was considered acceptable (Devellis, 2017).

#### Impact of Behaviors

To assist in validating the final measure, the impact of each item was examined, specifically the number and proportion of survivor participants who felt (moderately/very/extremely) scared and/or controlled due to each item (Table 4). The majority of those who had experienced psychological abuse during the last 12 months reported that the behaviors made them feel scared and controlled. For Factors 1 (Severe Psychological Abuse) and 2 (Coercive Emotional Abuse), participants described the behaviors as both fear-inducing and controlling, with only a slightly greater proportion favoring “control” as the predominant impact. On the other hand, the behaviors in Factors 3 (Restrictive Isolating Abuse) and 4 (Financial Abuse) were experienced by the majority of survivors as making them feel more controlled than scared.

**Table 4.** The Psychological Impact of Behaviors (*N* = 765).

Item	Proportion of participants who felt scared^a^ *n* (%)	Proportion of participants who felt controlled^b^ *n* (%)
Factor 1: Severe psychological abuse		
Punished or deprived the children to hurt me	74 (79.6)	79 (84.9)
Told me that they would kill me/my children	89 (84.0)	84 (79.2)
Threatened to take the children from me	93 (80.9)	93 (80.9)
Restricted access to essentials of daily living (e.g., food, phones, transport, housing, aids, visa/passport, or spiritual practice)	99 (77.3)	106 (82.8)
Threatened to harm family pets	92 (78.6)	96 (82.1)
Interfered with my health care (e.g., treatment for physical or mental health or alcohol and other drug issues)	87 (68.0)	101 (78.9)
Tried to damage my relationship with my child/ren	130 (71.8)	148 (81.8)
Factor 1 impact (mean)	77.2%	81.5%
Factor 2: Coercive emotional abuse		
Frightened me by screaming or breaking things	264 (81.0)	253 (77.6)
Threatened to destroy things	174 (78.0)	174 (78.0)
Put me down or humiliated me in front of others	180 (50.6)	241 (67.7)
Told me I imagined things or denied their own behavior	248 (53.3)	335 (72.0)
Drove erratically/dangerously when I or my child/ren were in the car	194 (84.7)	172 (75.1)
Factor 2 impact (mean)	69.5%	74.1%
Factor 3: Restrictive isolating abuse		
Controlled my day-to-day activities (e.g., where I go and what I do)	151 (61.9)	192 (78.7)
Kept track of where I was/who I was with (e.g., GPS tracking, monitoring social media, or phoning me)	146 (61.6)	183 (77.2)
Monitored my day-to-day activities or what I did with my time	184 (53.5)	231 (67.1)
Was jealous and possessive	201 (63.2)	250 (78.6)
Tried to keep me from socializing with family or friends	157 (57.1)	220 (80.0)
Factor 3 impact (mean)	59.5%	76.3%
Factor 4: Financial abuse		
Used our money/made important financial decisions without talking to me	157 (57.7)	205 (75.4)
Controlled my access to money	143 (67.1)	171 (80.3)
Took our/my money and made me worry about not having enough	132 (71.7)	152 (82.6)
Factor 4 impact (mean)	65.5%	79.4%

*Notes.* Base = all participants who had experienced abuse item in the last 12 months.

a,b Defined as behavior that made participants feel moderately, very, or extremely scared and/or controlled.

n.b. There was no missing data.

#### Scoring the PAR Scale

A preliminary scoring guide for the PAR Scale was developed based on consensus cut-off scoring by the research team. Each PAR behavioral item contained a possible frequency range of 0 to 5. Within each subscale, the possible range was: Severe Psychological Abuse (0–35), Coercive Emotional Abuse (0–25), Restrictive Isolating Abuse (0–25), and Financial Abuse (0–15). Subscales were not independent, and survivors could score across any or all. Individuals were categorized as experiencing psychological abuse if they scored ≥1 on the Severe Psychological Abuse items, ≥2 on the Coercive Emotional Abuse items, ≥2 on the Restrictive Isolating Abuse items, and/or ≥2 on the Financial Abuse items. The presence or absence of reaching the cut-off threshold in one or more PAR dimensions can be used to indicate the proportion of psychological IPV survivors in the last 12 months (prevalence). The scale should be used intact as a 20-item scale; the validity and reliability established for the PAR Scale as a whole may not generalize to subscales used in isolation or to the use of items selected from subscales. Three items in this measure are about children, and one is about pets. By adding an additional frequency label of “not applicable” next to only those items (i.e., *Not applicable*, *Not in the past 12 months*, *Once*, *A few times*, *Monthly*, *Weekly*, *Daily/almost daily*), people without children and/or pets can complete the scale in its current form using the same scoring conventions. The PAR Scale frequency measure can be used with or without asking about impact (scared/controlled). Further sensitivity and specificity testing may lead to future PAR Scale scoring changes.

## Discussion

The purpose of this study was to develop and preliminarily validate a new measure of psychological abuse victimization and impact that demonstrates acceptable reliability among a nationally representative sample of Australian women. The resulting PAR Scale is a concise, comprehensive, and multidimensional measure of victimization frequency and impact. Best practice steps were employed in development (Boateng et al., 2018). A review of the IPV literature identified existing measures of psychological abuse and informed an extensive list of items representing multiple psychological abuse types. Providing face and content validity, this list of items was reviewed, consolidated, and contemporized by academic and lived experience experts. Data was then collected from a sample of a magnitude sufficient to undertake exploratory factor analysis. The resulting four-factor solution generated acceptable internal reliability and validity (Devellis, 2017) and comprised four distinct dimensions.

### The Four Dimensions

The first dimension termed *Severe Psychological Abuse*, contains seven items describing threats of death or serious harm to the survivor, children, or pets, including being separated from children or trying to damage their relationship with children. Items within this factor reinforce the use of children as a psychological weapon that has been long reflected in the literature; rated by the highest number of survivor participants as making them feel scared and controlled (Fitzgerald et al., 2022; Follingstad, 2007; Tolman, 1989). Items about the use of power and coercion to restrict access to essentials of daily living and interfere with health care also loaded onto this factor, similar to the Severe Combined Abuse dimension of the Composite Abuse Scale (Hegarty et al., 2005). Common to all items in the Severe Psychological Abuse dimension is their fundamental importance and consequence to the survivor, making them powerful levers of fear and control over autonomy.

The second factor, titled *Coercive Emotional Abuse*, includes five tactics used by a perpetrator to degrade, shame, and hold the survivor responsible for the abuse or other perceived transgressions. Items describe screaming or breaking things, the threat of destroying things, dangerous driving, put-downs or humiliation in front of others, and being told the abuse was imagined. While many psychologically abusive behaviors can result in survivor subordination and an eroded sense of self, the items in this factor may play a unique role in these impacts (ANROWS, 2021). Notably, the items in this dimension constitute gaslighting and may engender a sense of impending harm, both well-documented features of coercion (Tolman, 1989; Winstok & Sowan-Basheer, 2015). Survivor participants found these items both frightening and controlling. Evidence suggests that threats may be used as part of a wider abuse campaign (Brown et al., 2021). The presence of threats in this and the Severe Psychological Abuse dimension may indicate a deliberate behavioral pattern intended to gain power or control over a partner (Aghtaie et al., 2018; Messing et al., 2020) and intensify the impact of abuse within a relationship (Brown et al., 2021).

The third factor, *Restrictive Isolating Abuse*, consists of five items that have in common their aim and/or effect of restricting and isolating the activities of a survivor. Items include controlling or monitoring day-to-day activities, restricting who they can socialize with, and behaving possessively toward the survivor. Confounded by a modern culture in which regularly “checking-in” with a partner via phone or other technology is a common occurrence, some items in this dimension may be couched in or obfuscated by their portrayal as acts of love, care, and concern (Aghtaie et al., 2018; Fernet et al., 2023). Instead, such restrictive and isolating behaviors can entrap a survivor and create dependence on the perpetrator (Dokkedahl et al., 2019; Stark, 2007; Stark & Hester, 2019; Tolman, 1989), thus functioning as part of a broader pattern of behavior to exercise control over a survivor’s activities, which is how survivor participants identified the behaviors made them feel.

The fourth factor, *Financial Abuse*, is comprised of three items that position money as a vehicle through which to enact constraint. The items in this factor are connected through their exploitation of a survivor’s financial resources or withholding the financial means needed for autonomy and escape (Kaittila et al., 2024; Stark & Hester, 2019). Financial abuse can be both coercive in nature and an act of control (Stark & Hester, 2019), and the emergence of this factor in the PAR Scale mirrors the consistency with which these behaviors appear in the literature (Fitzgerald et al., 2022; Follingstad et al., 2005; Kaittila et al., 2024; Tolman, 1999; WHO, 2021).

## Strengths and Limitations

The PAR Scale is a reliable and valid measure of psychological abuse, developed using best practice steps of scale development (Boateng et al., 2018). As a concise 20-item measure, the PAR Scale is less burdensome to complete than many other instruments (Rodenburg & Fantuzzo, 1993; Tolman, 1999). The scale is behaviorally based (Gomez-Fernandez et al., 2019), and captures the impact of a broad range of behaviors to advance our understanding of survivor experiences (Follingstad, 2007). By setting a threshold for psychological abuse, the scale may address a longstanding challenge in psychological abuse prevalence and association studies (Heise et al., 2019; WHO, 2021). The preliminary validation of the PAR Scale was undertaken with English-speaking women living in one high-income country, limiting the generalizability of the scale’s psychometric strength. The scale’s applicability for diverse survivors with different experiences and backgrounds is not yet known. Further, there are three items in the scale that pertain to children and one to pets, questions that will not be relevant for all survivors. Additional construct and predictive validity evidence would strengthen the scale’s utility.

## Future Directions

Many authors (Hegarty et al., 2005; Johnson et al., 2014; Winstok & Sowan-Basheer, 2015) contend that since different types of IPV are intertwined as a pattern of behaviors against an individual woman, to explore one particular type of abuse separately fails to acknowledge the holistic experience of an individual. Designed to better understand the range of psychologically abusive behaviors in a broader context of IPV, the PAR Scale was validated in a survey that included measures of all types of IPV (Australian Bureau of Statistics, 2017; Brown & Hegarty, 2021; Hegarty et al., 2005). In future papers, we will report the prevalence and associations between psychologically abusive behaviors and physical and sexual IPV. As several studies have found, relationships in which psychological abuse is present often include other types of abuse and violence (Mellar et al., 2024), thought of as coercive control (Lohmann et al., 2024). Such tactics and behaviors produce a micromanaged and rule-based environment that diminishes the survivors’ sense of self by causing fear, shame, and entrapment (Beckwith et al., 2023; Dokkedahl, 2021; Stark & Hester, 2019), enabling a perpetrator to maintain control (Sassetti, 1993). Thus, a comprehensive measure of coercive control that includes all types of abuse and has been validated with a diverse survivor sample is greatly needed.

## Conclusion

The PAR Scale is a comprehensive and multidimensional measure of psychological violence frequency and impact. The PAR Scale will enable researchers to study psychological abuse in-depth as a distinct type of IPV, making it a necessary addition to facilitate the measurement of IPV. IPV is best understood as a chronic, repeated experience characterized not by episodes of violence, but by an over-arching psychologically abusive climate that is created by the perpetrator to maintain control over his partner.
